# Longitudinally investigating patterns of maternal psychological distress in a South African birth cohort

**DOI:** 10.1186/s12889-025-24445-x

**Published:** 2025-10-08

**Authors:** Rae MacGinty, Nadia Hoffman, Nastassja Koen, Heather J. Zar, Dan J. Stein, Jennifer Pellowski

**Affiliations:** 1 Department of Psychiatry and Mental Health, University of Cape Town, Cape Town, South Africa; 2 South African Medical Research Council Unit On Risk and Resilience in Mental Disorders, Cape Town, South Africa; 3 Neuroscience Institute, University of Cape Town, Cape Town, South Africa; 4 Department of Pediatrics and Child Health, Red Cross War Memorial Children’s Hospital and South African Medical Research Council Unit On Child and Adolescent Health, University of Cape Town, Cape Town, South Africa; 5 Division of Epidemiology and Biostatistics, School of Public Health and Family Medicine, University of Cape Town, Cape Town, South Africa; 6 Department of Behavior and Social Sciences, Brown University School of Public Health, Providence, Rhode Island, USA

**Keywords:** Maternal psychological distress, Depression, Anxiety, Low and middle income, Longitudinal

## Abstract

**Background:**

Psychological distress, a broad construct that encompasses of a range of emotional difficulties—including depression and anxiety—is prevalent during pregnancy and postnatally with up to 25% of women experiencing psychological distress globally. However, this is mostly described in high income countries (HIC), with little data from low- and middle-income countries (LMICs). The aim of this study was to describe and determine latent trajectory classes of maternal psychological distress from pregnancy through five years postpartum in a LMIC setting.

**Methods:**

Data were used from women enrolled in the Drakenstein Child Health Study (DCHS), a birth cohort in South Africa. Women who had completed the Self-Report Questionnaire-20 items (SRQ-20) at two or more timepoints from pregnancy to 5 years postpartum, were included in the analysis. Latent class mixed modelling (LCMM) was used to generate latent trajectory classes of maternal psychological distress. Predictors, including trauma exposure, socio-economic indicators, substance use and pregnancy complications, of the latent classes were investigated using multinomial logistic regression.

**Results:**

In 973 women, four trajectory classes of maternal psychological distress were derived from pregnancy through 5 years postpartum, 1) persistent psychological distress symptoms, 2) antenatal symptoms only, 3) late postnatal onset of symptoms (post perinatal stage) and 4) low symptoms of psychological distress. Predictors of the persistent symptom class included early and recent trauma exposure, smoking during pregnancy, and gestational diabetes, whereas partner support was protective. Trauma exposure prior or during pregnancy was a predictor for the antenatal symptom class, while postnatal trauma exposure was associated with the late onset symptom class.

**Conclusion:**

Trajectories and predictors of maternal psychological distress were found to be similar to those seen in depression only and in HIC, which suggests there could be a degree of universality with regards to predictors of psychological distress. Trauma experienced by women was found to be a critical risk factor for psychological distress, as was maternal smoking or lack of partner support. There is an urgent need for social transformation surrounding gender-based violence and prevention and smoking cessation programs targeting women of child bearing age.

**Supplementary Information:**

The online version contains supplementary material available at 10.1186/s12889-025-24445-x.

## Introduction

It is estimated that up to 25% of pregnant women experience current psychological distress, a broad construct that encompasses depressive and anxiety symptoms [1]. Data from low- and middle-income countries (LMIC), suggests a higher prevalence of maternal depression and anxiety in these settings than in high income countries (HIC) [2, 3]. For example, a systematic review reported a significantly higher prevalence of depression in both antenatal (19.2% vs 9.2%) and postnatal (within 1 year postpartum) periods (18.7% vs 9.5%) in women in LMIC compared to HIC [2]. However, there are limited data on psychological distress in LMIC.

Maternal psychological distress during pregnancy may persist postpartum after the perinatal stage [4–8]. Furthermore, persistent maternal depression (or anxiety) has been linked with several adverse infant and early childhood outcomes [1, 9–29]. Studies in HIC, mostly focused on depression, have longitudinally investigated maternal depression or anxiety separately following the perinatal phase (> 2 years postpartum) and reported 3–6 trajectories of maternal depression post 2 years after delivery [5, 6, 8, 24, 30–34]. In addition, three distinct trajectories of maternal anxiety from pregnancy through to 5 years postpartum were derived in a Canadian population [8].

Fewer HIC studies have investigated longitudinal trajectories of maternal depression and/or anxiety (psychological distress), or the predictors of these symptoms over time [35, 36]. Five and four distinct latent trajectory classes were found in Australian and Norwegian populations respectively. Predictors for persistent psychological distress included history of depression, stressful life events, including stressors related to economic conditions [35, 36], while education and partner/family support providing protection again persistent psychological distress symptoms [36].

Few studies within LMIC, particularly in sub-Saharan Africa, have investigated trajectories of maternal depression or anxiety over time, particularly beyond 2–3 years postpartum [37–39]. Risk profiles in these environments may be different to HIC, as LMIC often have the additional burden of comorbidities, such as HIV, face socio-economic hardships and exposure to trauma or violence. Such environments may exacerbate psychological distress symptoms or alter patterns of symptoms over time in these settings. Therefore, in our analysis, we included a broad range of predictors spanning sociodemographic (e.g., age at enrollment, household income, educational achievement, self-reported employment, marital status), psychosocial (e.g., partner support, intimate partner violence, maternal childhood trauma, and stressful/traumatic life events), obstetric (e.g., gravida, gestational diabetes), behavioral (e.g., smoking, alcohol use), and health-related factors (e.g., HIV infection, history of asthma, hypertension, Body Mass Index (BMI)). These variables were selected based on prior evidence from both LMICs and HICs, where factors such as stressful life events, including trauma and economic stressors, and lack of partner or family support have been shown to influence the persistence or severity of psychological distress symptoms.

This study investigated the prevalence of psychological distress from pregnancy to 5 years postpartum, latent trajectories of symptom classes, and predictors associated with classes in a low-income community in South Africa.

## Methods

### Study design and setting

#### Setting

This study used data collected from the Drakenstein Child Health Study (DCHS) [40], a multidisciplinary birth cohort investigating the early life determinants of child health, including maternal mental health, in a peri-urban area in South Africa as previously described [40]. Participants were located in the Drakenstein area, a semi-urban area, in the town of Paarl approximately 60 km outside Cape Town, South Africa; the community, with an approximate population of 200 000, is stable, with low levels of immigration or emigration [40]. The population of the area are of low socio-economic status, live in informal housing or crowded conditions and have high levels of unemployment [40]. The local economy is based around commercial agriculture and light industry and more than 90% of the local population access health care in the public sector [40]. The Drakenstein area has a well-established, free primary health care system.

#### Participants & study design

Pregnant women were recruited from two primary health care clinics. Mothers were enrolled at 20–28 weeks’ gestation, between 1 March 2012 and 31 March 2015 [41]. Inclusion criteria were: 1) pregnant women 18 years or older, 2) attending one of the two primary clinics in the area and 3) intending to remain in the area for at least the next year [42]. Inclusion criteria were broad to ensure generalizability of results.

Consenting women completed study questionnaires and attended antenatal and postnatal follow-up visits at primary health care facilities. Biannual visits were conducted at the primary health care clinics, and the annual visits were conducted at Paarl Hospital. This sub-study uses data collected during pregnancy through 5 years (60 months) postpartum.

The DCHS study was approved by the Faculty of Health Sciences, Human Research Ethics Committee, University of Cape Town (401/2009), and by the Western Cape Provincial Health Research committee (2011RP45).

### Measures

#### Maternal psychological distress

Psychological distress was measured by the Self-Report Questionnaire- 20 items (SRQ-20), a validated, World Health Organization (WHO) endorsed, screening tool that was designed to screen psychological distress in LMICs based on 20 yes/no questions [43–47]. A value of 1 was assigned to each answer of “yes” and 0 to “no”, and the total score determined, with a maximum score of 20 and a score of 8 or more indicating “above threshold” for the presence of psychological distress symptoms. The total score was used in the analysis in this study. The SRQ-20 was completed once antenatally and postnatally at 10 weeks, 6, 12, 18, 24, 36, 48 and 60 months post-delivery.

#### Maternal psychosocial exposures & substance use 

The presence of intimate partner violence (IPV) was assessed using adapted measures from the WHO multi-country study [48] and the Women's Health Study in Zimbabwe [49]. A total of 17 questions were asked, covering emotional (5 items), physical (6 items), and sexual abuse (4 items), with most items rated on a Likert scale (ranging from 1 (“never”) to 4 (“many times) and one dichotomous item per domain assessing recent exposure (past 12 months). Two additional open-ended questions asked participants how the questions made them feel and invited further comments. Total scores are calculated for each domain of IPV: Emotional, physical or sexual abuse. Exposure to IPV was calculated (any items > = 2) as any recent experiences of physical, emotional or sexual abuse by their partner (within 12 months of completing questionnaire).

Maternal childhood trauma exposure was measured using The Childhood Trauma Questionnaire (CTQ) – Short Form which identifies the presence of childhood abuse and neglect [50]. Participants rated each item on a Likert scale from 1 (“never true”) to 5 (“very often true”). The total score from all subscales was used, and participants were categorized as either above (Total Score > 36) or below (Total Score ≤ 36) the threshold for experiencing childhood trauma based on established guidelines [50]. The total score of maternal childhood trauma was included in the final analysis, while the dichotomous variable was used for reporting purposes.

Stressful life events in the past 12 months were reported using the World Mental Health Life Events Questionnaire [51]. Each event was scored as either experienced (“1”) or not experienced (“0”), with a maximum total score of 17 indicating higher exposure to life events. The total score of stressful life events was included in this analysis.

Smoking tobacco and drinking alcohol were self-reported using the Alcohol, Smoking and Substance Involvement Screening Test (ASSIST) [52]. For reporting purposes tobacco use and alcohol use were categorized as either any use or no use. Total scores for alcohol consumption were included in the predictor analysis. In conjunction with the ASSIST, the women also self-reported (dichotomous question of yes or no) smoking during pregnancy, and postnatally.

The use of these measures has also been previously described [42]. Each of these measures, except the CTQ, was captured antenatally and postnatally at 10 weeks, 6, 12, 18, 24, 36, 48 and 60 months post-delivery. The CTQ was administered antenatally only. For the purpose of this study, postnatal exposure to IPV, stressful life events and substance use were considered annually at 12, 24, 36, 48 and 60 months post-delivery.

The DCHS has protocols for referring participants with mental health conditions to the public health system in the study area.

#### Maternal characteristics

Additional maternal characteristics also collected included age at enrolment, HIV status, determined through testing during pregnancy, and any illnesses during pregnancy, including gestational diabetes, hypertension, anemia or asthma, which were either self-reported or diagnosed by a medical professional antenatally. In addition, anthropometric measures including weight and height were measured during pregnancy and were used to calculate Body Mass Index (BMI). Birth planning, marital status as well as support from the father/partner reported by the women on a Likert scale (where 1 = ”Not at all supportive”; 2 = ”Slightly supportive”; 3 = ”Moderately supportive”; 4 = ”Considerably supportive”; 5 = “Extremely supportive”) and socio-economic status (SES) were also collected through adapted from items used in the South African Stress and Health Study (SASH) [51]. The SES data included self-reported household factors such as household income, number of assets and household crowding, and self-reported maternal demographics such as educational achievement and employment, where the typical occupations included seasonal work on farms, factory work, retail, healthcare and teaching.

#### Child birth measures

Data collected for child at delivery include child sex; gestational age at delivery and anthropometric measures including birth weight. Anthropometric z-scores were generated using World Health Organization (WHO) growth standards [53] at birth for full-term children. Fenton Growth chart for premature birth [54, 55] was used to generate z-scores at birth for premature children.

### Statistical analysis

Data analysis was done using Stata (Stata Statistical Software: Release 18. College Station, TX: StataCorp LLC) and R (R Core Team, 2021, version 4.1.2). Frequencies, proportions, means and standard deviations were reported where applicable. Chi-square, Fisher Exact Test, and T-tests were used where appropriate for exploratory purposes to identify statistical differences among categorical and continuous variables respectively.

Confirmatory factor analysis (CFA) was performed to determine if there were any clustering of questions in the SRQ-20, but only one factor was observed, suggesting that the whole questionnaire should be used as a cohesive unit. Cronbach’s Alpha (α = 0.88) was also used to validate the internal reliability of the SRQ-20 across all time points. To test the assumption of data missing completely at random (MCAR), Little's MCAR was used.

Latent Class Mixed Modelling (LCMM) was used to identify longitudinal trajectories of maternal psychological distress scores over the study period, using the ‘*lcmm’* package in R. LCMM inference is based on maximum likelihood, thus estimates of the dependent variable are robust to missing data. In addition, LCMM incorporates random effects to account for intra-participant correlation over time. A linear link was specified, as the outcome of interest was continuous. The optimal number of trajectory groups was determined using Bayesian Information Criteria (BIC). To maintain interpretability and clinical relevance, the maximum number of groups considered was six; six groups was also the maximum number of groups determined in prior research of depression trajectories [5, 6, 8, 24, 30–34]. To establish the number of groups, each group was initially modeled using cubic polynomial terms. The optimal functional form (linear, quadratic, or cubic) was then determined for each group using BIC. Posterior probabilities and entropy were then used to investigate the stability of the final model and assess the fit. Finally, K-fold cross validation, with 5 folds, using the ‘*modelr’* package in R*,* was used to examine the reproducibility of the final model.

In addition, multinominal logistic regression was used to identify predictors of latent symptom classes. Each participant was assigned to a class based on the class with the maximum posterior probability of class membership. The largest class, the low symptom class, was used as the reference category group. The predictors were explored using a complete case analysis and after imputing missing data using multiple imputation by chained equations (MICE). Predictive mean matching (PMM), logit and multinomial logit models were used to impute data for continuous, binary and categorical variables respectively. The missingness was assumed to be at random and 20 imputations were performed, with Rubin’s Rule pooling the estimates across the imputed datasets. The multinomial logistic regression models and multiple imputation were produced using Stata. Results from complete case and imputed datasets produced similar conclusions, thus we reported results from the imputed datasets. In addition, diagnostic checks, including variance inflation factor (VIF) to test for multicollinearity, were performed to ensure that the models were valid.

## Results

A total of 1137 mothers, with 1143 live births (3 sets of twins and one set of triplets), were enrolled in the DCHS. There were *n* = 981 children and 977 mothers in the DCHS at 5 years after delivery (162 children terminated), supplementary Fig. 1. In this study, women with at least two visit completions over the 5-year period were considered, thus 973 women (and 977 children) were included in the final analysis. Characteristics between those included and excluded were similar; however, a slightly higher BMI and prevalence of smoking during pregnancy (29.6% vs 21.3%) was observed in those included in the analysis. In addition, there were more participants in lowest household income category and lower educational achievements in the included group.

Women included had an average age of 26.7 years (Table 1); 211 (21.7%) were living with HIV. Few women had complications from asthma (1.2%), gestational diabetes (1.3%) or hypertension (4.3%) during pregnancy, although 22% (*n* = 211) had anemia; 30% (*n* = 288) self-reported smoking during pregnancy. Most women were single (60%), however 80% of the women reported having some support from their partner or the father of the child. One quarter (*n* = 252) of the women were unemployed at the time of enrolment and 88% were living in households that earned less than 5000 ZAR [$280] per month. In addition, more than 60% of the women had not completed secondary schooling.

**Table 1:** Characteristics of mothers at enrolment

*Maternal characteristics*	*N* = 973
*Socio-demographic characteristics*	
Age enrolment [Mean (SD)]	26.72 (5.73)
BMI at enrolment [Mean (SD)]	28.36 (6.43)
Household income per month: < 1000 ZAR [$55]	342 (35.2%)
1000–5000 ZAR	511 (52.5%)
> 5000 ZAR [$280]	120 (12.3%)
Self-reported Employment	252 (25.9%)
Educational achievement: Did not complete secondary	613 (63%)
Completed secondary or above	360 (37%)
Marital status: Single	587 (60.3%)
Married	386 (39.7%)
Partner support: No support	40/866 (4.6%)
Slight-moderate support	135/866 (15.6%)
Considerable-extreme support	691/866 (79.8%)
Gravida [Mean (SD)]	1.12 (1.09)
*Infections/illnesses during pregnancy*	
HIV infection	211 (21.7%)
Gestational diabetes during pregnancy	13 (1.3%)
Asthma during pregnancy	12 (1.2%)
Hypertension during pregnancy	42 (4.3%)
Anemia during pregnancy	211 (21.7%)
*Substance use*	
Smoking during pregnancy	288 (29.6%)
Any alcohol use	141/862 (16.4%)
*Psychosocial measures*	
Psychological distress (above threshold: score ≥ 8)	175/869 (20.1%)
IPV (any recent physical, emotional, or sexual IPV)	295/869 (34.0%)
Maternal childhood trauma (above threshold: score ≥ 37)	296/869 (34.1%)
Traumatic life events (*n* = 867) [Mean (SD)]	1.94 (2.22)

*SD* Standard deviation, *BMI* Body mass index, *HIV* Human Immunodeficiency Viruses, *IPV* Intimate partner violence

High levels of exposure to traumatic events were reported antenatally, with 34% of the women reporting recent IPV (*n* = 295/869), and 34% (*n* = 296/869) experiencing maternal childhood trauma. On average, 1.9 traumatic life events per women were reported, supplementary Table 2.

The highest prevalence of psychological distress was observed antenatally (20%, defined by a score ≥ 8), Table 2. The overall prevalence of psychological distress steadily declined after birth, however, there was a slight increase at 36 (11%) and 48 (10%) months postpartum, with 58 (7%) of women reporting symptoms at the 60 months, Table 2. Little’s MCAR test was used to assess whether completion of the SRQ-20 was missing completely at random (MCAR). The test was non-significant (*p* = 0.28), indicating that the null hypothesis of data being MCAR could not be rejected. This supports the use of LCMM, which assumes data are missing at random.

**Table 2:** Summary statistics of maternal psychological distress over time in mothers included in analysis

	**During pregnancy**	**Postpartum**							
	**Antenatal visit**	**10 weeks**	**6 months**	**12 months**	**18 months**	**24 months**	**36 months**	**48 months**	**60 months**
N completed SRQ-20	869	636	615	745	660	747	787	873	829
Above threshold*	175 (20.1%)	63 (9.9%)	58 (9.4%)	70 (9.4%)	52 (7.9%)	61 (8.2%)	87 (11.1%)	87 (10.0%)	58 (7.0%)
Mean (SD)	4.43 (3.82)	2.69 (3.33)	2.51 (3.51)	2.30 (3.53)	2.18 (3.51)	2.03 (3.41)	2.53 (3.72)	2.48 (3.69)	2.09 (3.23)

*SRQ-20* Self-report questionnaire – 20 items, *SD* Standard deviation

^*^Score ≥ 8 on SRQ-20

**Fig. 1:**
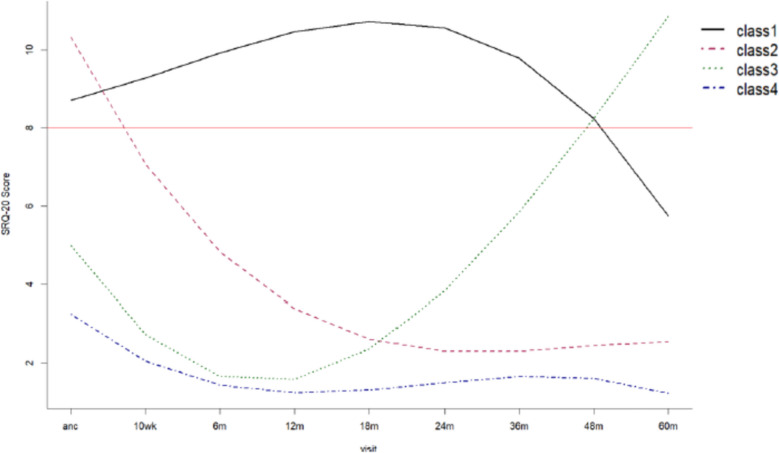
Latent class mixed model depicting trajectories of psychological distress from antenatal to 60 months postpartum visits. Four classes identified: Class 1 represents the cumulative psychological distress symptoms group (*n* = 69); Class 2 represents antenatal symptoms only (*n* = 77); Class 3 represents late onset psychological distress (*n* = 38); Class 4 represents no symptoms (*n* = 789). The red reference line (SRQ-20 = 8) reflects the cut-off score indicative of above threshold for psychological distress

Using LCMM, four latent classes of psychological distress trajectories were derived. These consisted of those with persistent symptoms over the 5-year period, but with decreasing symptoms from 4 years postpartum (class 1, *n* = 77); those reporting antenatal symptoms with decreasing symptoms postpartum (class 2, *n* = 69); those reporting few symptoms antenatally but increasing symptoms postpartum or late onset post the perinatal stage (class 3, n = 38); and those with little to no symptoms reported across the 5 years (class 4, n = 789), Fig. 1.

Multinomial logistic regression was used to determine predictors of the latent classes of maternal psychological distress. The regression model was run using a complete case approach (supplemenatry Table 3, 4) and after imputating missing data (Table 3), with similar results. Predictors for the latent classes were captured prior or during pregnancy.

**Table 3:** Prenatal Predictors of latent tracjectory classes of psychological distress after multiple imputation

	**Class 1 vs Class 4**	**Class 2 vs Class 4**	**Class 3 vs Class 4**
	Adjusted RRR (95% CI)*	Adjusted RRR (95% CI)*	Adjusted RRR (95% CI)*
Age at enrolment	0.99 (0.92; 1.07)	**0.91 (0.85; 0.98)**	1.01 (0.93; 1.10)
BMI	0.99 (0.94; 1.04)	1.01 (0.97; 1.06)	0.98 (0.92; 1.04)
Houeshold income: < 1000 ZAR	Reference	Reference	Reference
1000–5000 ZAR	0.62 (0.32; 1.18)	0.70 (0.38; 1.29)	0.58 (0.26; 1.30)
> 5000 ZAR	0.80 (0.29; 2.19)	1.05 (0.44; 2.50)	0.80 (0.25; 2.62)
Self-reported Employment	0.77 (0.36; 1.63)	1.35 (0.71; 2.55)	1.04 (0.44; 2.48)
Educational achievement:			
Did not complete secondary	Reference	Reference	Reference
Completed secondary or above	1.18 (0.61; 2.28)	0.80 (0.44; 1.45)	0.76 (0.35; 1.68)
Marital status: Single	Reference	Reference	Reference
Married/co-habiting	1.28 (0.66; 2.47)	1.53 (0.82; 2.84)	1.41 (0.63; 3.14)
Partner support: No support	Reference	Reference	Reference
Slight/moderate support	0.59 (0.19; 1.82)	0.33 (0.11; 1.05)	1.19 (0.13; 11.24)
Considerable/extreme support	**0.30 (0.11; 0.80)**	**0.35 (0.13; 0.92)**	1.24 (0.15; 9.95)
Gravida	1.26 (0.90; 1.77)	1.14 (0.81; 1.61)	0.96 (0.62; 1.49)
HIV infection	0.53 (0.21; 1.32)	1.33 (0.68; 2.61)	1.96 (0.90; 4.27)
Gestational diabetes	**7.03 (1.43; 34.50)**	-	3.46 (0.37; 32.58)
Asthma	1.63 (0.18; 14.74)	1.13 (0.12; 10.29)	-
Hypertension	2.28 (0.70; 7.39)	1.65 (0.45; 5.99)	0.72 (0.09; 5.87)
Anaemia	0.49 (0.21; 1.12)	1.07 (0.58; 1.99)	0.59 (0.24; 1.47)
Smoking during pregnancy	**1.96 (1.05; 3.65)**	0.87 (0.48; 1.56)	1.10 (0.48; 2.48)
Alcohol**	1.02 (0.99; 1.06)	1.03 (0.99; 1.06)	1.02 (0.98; 1.07)
IPV	1.53 (0.84; 2.80)	1.50 (0.85; 2.66)	0.61 (0.26; 1.40)
Maternal childhood trauma**	**1.05 (1.03; 1.07)**	**1.03 (1.01; 1.05)**	**1.03 (1.00; 1.06)**
Traumatic Life events**	**1.27 (1.12; 1.43)**	**1.32 (1.18; 1.48)**	1.12 (0.94; 1.33)

*RRR* Relative risk ratio, *CI* Confidence interval, *BMI* Body mass index, *HIV* Human Immunodeficiency Virus, *IPV* Intimate partner violence, *Class 1* Persistent psychological distress symptoms, *Class 2* Antenatal symptoms only, *Class 3* Late onset psychological distress, *Class 4* No psychological distress

^*^n = 973

^**^Total scores for psychosical measures used

Gestational diabetes (adjusted risk ratio (RR) = 7.03, 95% Confidence interval (CI): 1.43; 34.50), antenatal smoking (adjusted RR = 1.96, 95% CI: 1.05; 3.65), prior childhood trauma (ajusted RR = 1.05, 95% CI: 1.03; 1.07) or a traumatic life event (adjusted RR = 1.27, 95% CI: 1.12; 1.43) all increased the risk of persisent maternal psychological distress (class 1) vs no psychological distress (class 4) in the adjusted model, Table 3. In contrast, considerable partner support decreased the risk of persistent psychological distress (adjusted RR: 0.30, 95% CI: 0.11; 0.80) over the study period. IPV exposure or alcohol use were also found to be independent predcitors of the persistent psychological distress class (class 1) compared to the low symptom class (class 4), but these assocaitions fell away in the adjusted model, supplementary Table 4. No socio-economic covariates were associated with persistent psychological distress.

Maternal childhood trauma (adjusted RR: 1.03, 95% CI: 1.01; 1.05) or prior traumatic experiences (adjusted RR: 1.32, 95% CI: 1.18; 1.48) were also predictive of antenatal psychological distress (class 2) vs the low symptoms class (class 4), Table 3. Considerable partner support (adjusted RR: 0.35, 95% CI: 0.13; 0.92) and maternal age (adjusted RR: 0.91, 95% CI: 0.85; 0.98) were found to be protective against antenatal psychological distress. Again, antenatal smoking, IPV or alcohol use were strong independent predictors, but these were not significant in the adjusted model.

The only predictor of late onset psychological distress (class 3) was maternal childhood trauma. Maternal HIV infection was a predictor of this class in the complete case analysis, supplemtary Table 4, but was not significant after imputing missing data.

In addition, postnatal risk factors associated with the latent psychological distress classes was also investigated. IPV at both 12 and 24 months postpartum, as well as any traumatic life events reported at all postnatal annual visits were strongly associated with the persistent group (class 1) vs no exposure (class 4), supplementary Table 5. IPV reported at all annual postnatal visits, and any traumatic life events reported from the 36 month postpartum visit onwards were all associated with the late onset psychological distress class (class 3), supplementary Table 5. Further, maternal HIV infection remained strongly associated with the late onset class at both the 12 and 24 month postpartum visits, but thereafter was no longer a significant factor. Child sex, preterm delivery and birth weight were not associated with any of the classes postnatally.

## Discussion

Maternal psychological distress was highly prevalent from pregnancy through 5 years postpartum in a low income area of South Africa, although symtoms of psychological distress declined over time (20% antenatally to 7% at five years postpartum). Four trajectory classes of maternal psychological distress of symptoms were derived: persistent symptoms over the study period; an antenatal symptom only class; a late onset of symptoms class, with increasing symptoms from 24 months postpartum; and those with minimal symptoms over the whole study period. From classes 1 through 3, it was observed that 19% of women displayed signs of transient or chronic symptoms over the study period. Both early and recent (within 12 months) Trauma exposure or antenatal maternal smoking were strongly predictive of persistent maternal psychological distress class compared to the low symptom class. Further, gestational diabetes was a predictor of the persistent symptom class, while partner support was protective against persistent symptoms of psychological distress.

To our knowledge, this is the first study that has investigate longitudinal trajectories of maternal psychological distress in a LMIC. Two previous studies, both of which were conducted in HIC, investigated trajectories of postnatal maternal pyschological distress, finding a similar number of distinct trajectory classes: 5 classes in Australia [35] and 4 classes in Norway [36]. The strongest risk factors for persistent psychological distress in these studies, included history of depression, experiencing stressful life events [35, 36], and tobacco use [35]; while partner or social support was protective against psychological distress [36]. In a prior study conducted in the DCHS focusing on depression trajectories from pregnancy to 18 months postpartum, stressful life and events and tobacco use were found to predict severe depression [37]. Taken together, then, risk and resilience factors/mechanisms for distress and depression seem to have a degree of universality.

Some additional findings in the current study should be highlighted. The association between gestational diabetes and the persistent psychological distress class (vs the low symptom class) is notable. Gestational diabetes had previously been found to be a strong predictor of persistent postpartum maternal depression [5, 56–60], but the association between gestational diabetes and distress or depression is not well understood. Concerns about having diabetes represents one casual mechanism [61], but other causal mechanisms or common risk factors, such as low socio-economic status may play a role. Notably, in the current study, no socio-economic factors were associated with any of the classes.

Antenatal smoking proved to be a strong predictor of persistent psychological distress, which is consistent with prior evidence, where a strong link between maternal smoking and antenatal and postnatal depression or distress has been observed [35, 36, 62]. Antenatal smoking prevention and cessation programmes could provide help reduce the burden of persistent psychological distress during and after pregnancy.

Trauma exposure, including maternal childhood trauma and traumatic stressful life events, were predictive of the persistent, antenatal and late onset psychological distress symptoms classes. Trauma exposure is highly prevalent in the study participants, with more than one third of the women reporting IPV and maternal childhood trauma exposure during pregnancy. IPV was independently associated with the persistent and antenatal maternal psychological distress, but this association fell away in the adjusted models. However, addressing such trauma exposures is critical to reducing the burden of pychological distress. In particular, addressing social/structural factors that increase rates of trauma exposure, such as gender based violence, seems key. Partner support, particularly father involvement during and after pregnancy, has shown promise in improving maternal mental health and reducing domestic violence. For example, a study in Soweto, South Africa found that greater father involvement was associated with reduced maternal depression [63], while a systematic review of father-inclusive interventions, including community-based or family centered education programs, in LMICs reported benefits for maternal well-being, couple relationships, and early child outcomes [64]. A number of interventions have already been established in LMIC to combat gender based violence and maternal mental health concerns. For example, parenting interventions such as Parenting for Lifelong Health (PLH), which engages both mothers and fathers, have been shown to reduce child maltreatment and improve caregiver mental health in LMICs [65]. Additionally, preconception and adolescent-focused interventions, such as the Skhokho Supporting Success program in South Africa, integrate gender-based violence prevention and promote healthy relationships and mental well-being among adolescents [66]. Finally, a pilot randomised controlled trial in Malawi demonstrated that the adaptation of the Friendship Bench—a problem-solving therapy delivered by trained community health workers—was effective in reducing symptoms of depression among perinatal women [67].These interventions highlight scalable, culturally adapted approaches that integrate mental health care and violence prevention, offering promising models for broader implementation in similar contexts.

There are several limitations to the current study. Firstly, the maternal psychosocial exposures, were self-reported, thus there may be bias in reporting symptoms in the cohort. Despite the possibility of under-reporting, the high burden of psychological distress and trauma exposure highlights the urgent need for intervention. The decline in psychological distress over time, as also reported by Woody et al. (2017), may also reflect underreporting due to the stigma surrounding maternal mental health, particularly when using self-reported measures. In contrast, other studies [68], have observed an increase in symptoms from the antenatal to postnatal period. These differing patterns may be explained by variations in study populations, assessment tools, or the timing of symptom measurement. Secondly, in our multinomial logistic regression analyses, some classes had small sample sizes, and may be underpowered to detect all relevant predictors. Although several predictors were signficant, the true magnitude of the effect of these on class classification is unclear. Thirdly, including multiple trauma-related exposures in the model may introduce complexity due to overlapping constructs and temporal proximity. This may reflect shared variance, potential confounding, or that later trauma may act as a mediator of earlier trauma. However, as we are not testing a specific exposure-outcome relationship, our focus is on identifying key predictors rather than disentangling causal pathways. Finally, there were some differences between those included in these analyses and the full population-based sample. Those living in lower income households (< 1000 ZAR [$55]) were more likely to attend more visits and complete the SRQ-20 assessment, and thus, be included in this sample. However, his suggests that the more at-risk women remained in the study.

There are, however, several strengths to the study. This is the first study, to our knowledge, that has investigated longitudinal psychological distress trajectories in a large cohort of women antenatally and postnatally in LMIC. The robustness of LCMM also provides a strength as random effects can be incorporated into the model and the maximum likelihood method of estimation accounts for missing data, assuming the data is random. The prospective, longitudinal nautre of data collection in the DCHS, as well as the strong retention of participants (85% retention post 5 years), make this a particularly strong study, and one of the largest birth cohorts in LMIC. In addition, the assessment of trauma exposure (including maternal childhood trauma and IPV exposure) represents a significant strength of this study, as such measures are rarely included alongside depressive and anxiety symptoms in perinatal or maternal populations. Finally, this study recruited a population-based clinic sample, which adds to the generalizability of these results to peri-urban areas, particularly in LMIC.

## Conclusion

Taken together, a high prevalence of maternal psychological distress was observed in this South African setting, and although prevalence declined over time, a high burden of psychological distress persisted. Four distinct trajectory class of maternal psychological distress were identified from pregnancy through 5 years postpartum. Trauma exposure, maternal smoking or gestational diabetes were strong predictors of persistent psychological distress, whereas partner support was protective against persistent psychological distress. Future research of trajectories of maternal psychological distress should be conducted in rural and other LMIC settings to confirm the findings. This research illustrates the need for prevention and smoking cessation programs for women of child bearing age, and highlights the important role of trauma experienced by women as a critical risk factor for psychological distress. Social transformation, including gender-based violence, is need of urgent attention.

## Supplementary Information

Supplementary Material 1: Supplementary figure 1. Flow chart of enrolment and terminations in Drakenstein Child Health Study (DCHS)*.*

Supplementary Material 2: Supplementary figure 2. Heat map depicting total SRQ-20 scores (orange), as well as missed visits (grey) over study period

Supplementary Material 3: Supplementary Table 1. Characteristics of included vs excluded participants. Supplementary Table 2. Summary statistics of maternal psychosocial exposures over time in mothers included in analysis. Supplementary Table 3. Unadjusted multinomial logistic regression model of predictors of the latent tracjectory classes of psychological distress using a complete case approach. Supplementary Table 4. Adjusted multinomial logistic regression model of Predictors of the latent tracjectory classes of psychological distress using a complete case approach. Supplementary Table 5. Postnatal risk factors associated with the latent tracjectory classes of psychological distress using a complete case approach

## Data Availability

An anonymised, de-identified version of the dataset can be made available on request to allow all results to be reproduced. All requests should be directed to Heather Zar (heather.zar@uct.ac.za), the Principal Investigator of the Drakenstein Child Health Study.

## Abbreviations

ASSIST: Alcohol, smoking and substance involvement screening test
BIC: Bayesian Information Criteria
BMI: Body Mass Index
CFA: Confirmatory factor analysis
CI: Confidence interval
CTQ: Childhood trauma questionnaire
DCHS: Drakenstein Child Health Study
HIC: High income countries
HIV: Human immunodeficiency virus
IPV: Intimate partner violence
LCMM: Latent class mixed modelling
LMIC: Low and middle income countries
LRTI: Lower respiratory tract infections
MCAR: Missing completely at random
MICE: Multiple imputation by chained equations
PMM: Predictive mean matching
RRR: Relative Risk ratio
SASH: South African Stress and Health Study
SES: Socio-economic status
SRQ-20: Self-Report Questionnaire-20 items
VIF: Variance inflation factor
WHO: World Health Organization
ZAR: South African Rands
