# Supplementary figures and images for: Longitudinally investigating patterns of maternal psychological distress in a South African birth cohort
Source: BMC Public Health. 2025 Oct 8;25:3409. doi: 10.1186/s12889-025-24445-x (PMC12505592; doi:10.1186/s12889-025-24445-x)

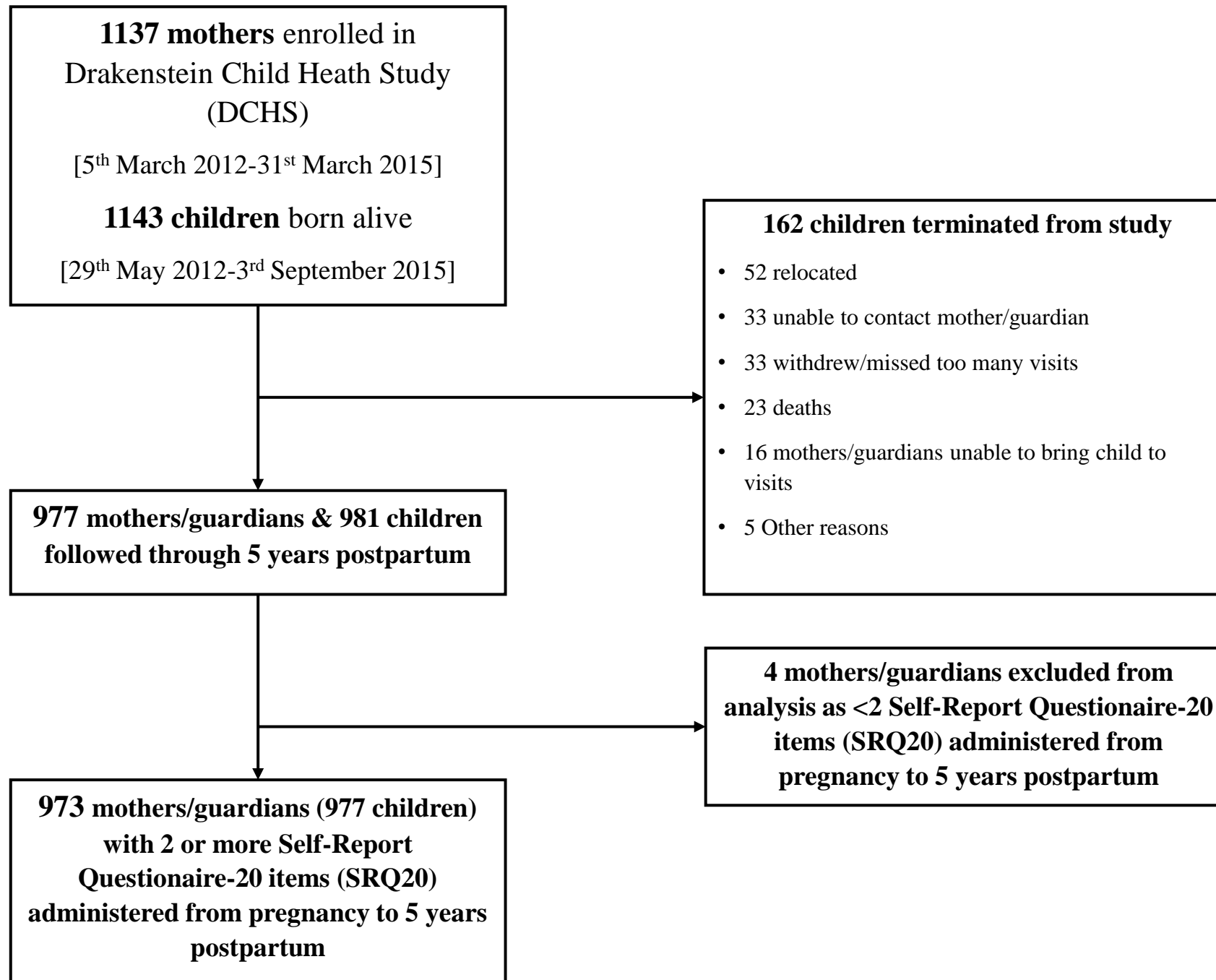

Supplement: Supplementary file 1 — Supplementary Material 1: Supplementary figure 1. Flow chart of enrolment and terminations in Drakenstein Child Health Study (DCHS). [file 12889_2025_24445_MOESM1_ESM.pdf]

Children

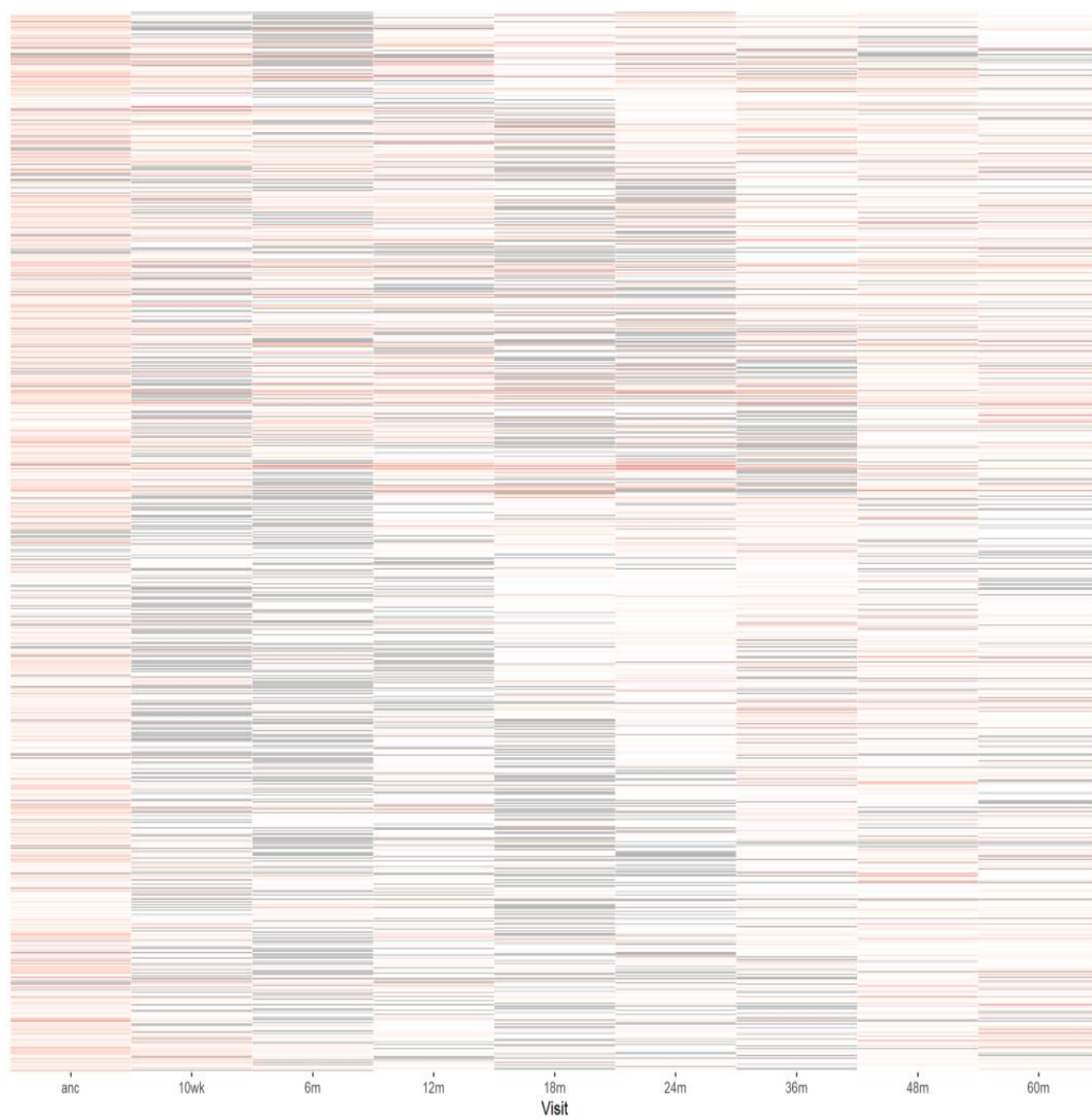

Supplement: Supplementary file 2 — Supplementary Material 2: Supplementary figure 2. Heat map depicting total SRQ-20 scores (orange), as well as missed visits (grey) over study period [file 12889_2025_24445_MOESM2_ESM.pdf]
