# Supplementary material for: Longitudinally investigating patterns of maternal psychological distress in a South African birth cohort
Source: BMC Public Health. 2025 Oct 8;25:3409. doi: 10.1186/s12889-025-24445-x (PMC12505592; doi:10.1186/s12889-025-24445-x)
Supplement: Supplementary file 3 — Supplementary Material 3: Supplementary Table 1. Characteristics of included vs excluded participants. Supplementary Table 2. Summary statistics of maternal psychosocial exposures over time in mothers included in analysis. Supplementary Table 3. Unadjusted multinomial logistic regression model of predictors of the latent tracjectory classes of psychological distress using a complete case approach. Supplementary Table 4. Adjusted multinomial logistic regression model of Predictors of the latent tracjectory classes of psychological distress using a complete case approach. Supplementary Table 5. Postnatal risk factors associated with the latent tracjectory classes of psychological distress using a complete case approach [file 12889_2025_24445_MOESM3_ESM.docx]

|  | **Total enrolment** | **Included** | **Excluded** | **P-value** |
| --- | --- | --- | --- | --- |
| ***Maternal characteristics*** | **N=1137** | **N=973** | **N=164** |  |
| *Socio-demographic characteristics* |  |  |  |  |
| Age enrolment [Mean (SD)] | 26.58 (5.68) | 26.72 (5.73) | 25.79 (5.33) | 0.053 |
| BMI at enrolment [Mean (SD)] | 28.20 (6.36) | 28.36 (6.43) | 27.02 (5.69) | **0.023** |
| Household income per month: <1000 ZAR [$55] | 385 (33.9%) | 342 (35.2%) | 43 (26.4%) | **<0.001** |
| 1000-5000 ZAR | 592 (52.1%) | 511 (52.5%) | 81 (49.7%) |  |
| >5000 ZAR [$280] | 159 (14%) | 120 (12.3%) | 39 (23.9%) |  |
| Self- reported Employment | 307 (27%) | 252 (25.9%) | 55 (33.5%) | **0.042** |
| Educational achievement: Did not complete secondary | 692 (50.9%) | 613 (63%) | 79 (48.2%) | **<0.001** |
| Completed secondary or above | 445 (39.1%) | 360 (37%) | 85 (51.8%) |  |
| Marital status: Single | 679 (59.7%) | 587 (60.3%) | 92 (56.1%) | 0.307 |
| Married | 458 (40.3%) | 386 (39.7%) | 72 (43.9%) |  |
| Partner support: No support | 45/991 (4.5%) | 40/866 (4.6%) | 5/125 (4%) | 0.284 |
| Slight-moderate support | 148/991 (14.9%) | 135/866 (15.6%) | 13/125 (10.4%) |  |
| Considerable-extreme support | 798/991 (80.5%) | 691/866 (79.8%) | 107 (85.6%) |  |
| Gravida [Mean (SD)] | 1.10 (1.09) | 1.12 (1.09) | 1.00 (1.04) | 0.193 |
| *Infections/illnesses during pregnancy* |  |  |  |  |
| HIV infection | 244 (21.5%) | 211 (21.7%) | 33 (20.1%) | 0.652 |
| Gestational diabetes during pregnancy | 14 (1.2%) | 13 (1.3%) | 1 (0.6%) | 0.435 |
| Asthma during pregnancy | 15 (1.3%) | 12 (1.2%) | 3 (1.8%) | 0.536 |
| Hypertension during pregnancy | 55 (4.8%) | 42 (4.3%) | 13 (7.9%) | **0.046** |
| Anemia during pregnancy | 238 (20.9%) | 211 (21.7%) | 27 (16.5%) | 0.128 |
| *Substance use* |  |  |  |  |
| Smoking during pregnancy | 323 (28.4%) | 288 (29.6%) | 35 (21.3%) | **0.030** |
| Any alcohol use | 164/986 (16.6%) | 141/862 (16.4%) | 23/124 (18.6%) | 0.540 |
| *Psychosocial measures* |  |  |  |  |
| Psychological distress (above threshold: score≥8) | 202/995 (20.30%) | 175/869 (20.1%) | 27/126 (21.4%) | 0.736 |
| IPV (any recent physical, emotional, or sexual IPV) | 334/994 (33.6%) | 295/869 (34.0%) | 39/125 (31.2%) | 0.543 |
| Maternal childhood trauma (above threshold: score≥37) | 343/995 (34.5%) | 296/869 (34.1%) | 47/126 (37.3%) | 0.475 |
| Traumatic life events [Mean (SD)] | 1.96 (2.22), n=992 | 1.94 (2.22), n=867 | 2.06 (2.22), n=125 | 0.575 |
| ***Child birth characteristics*** | ***N=1143*** | ***N=977*** | ***N=166*** |  |
| Sex - female | 557 (48.7%) | 479 (49.0%) | 78 (47.0%) | 0.627 |
| Preterm delivery (<37 Weeks gestation) | 192 (16.8%) | 154 (15.8%) | 38 (22.9%) | **0.023** |
| Birth weight z-score [Mean (SD)] | -0.27 (1.06) | -0.28 (1.08) | -0.20 (0.90) | 0.375 |

**Supplementary Table 1.** Characteristics of included vs excluded participants.

SD: Standard deviation; BMI: Body mass index; HIV: Human Immunodeficiency Viruses; IPV: Intimate partner violence

|  | **During pregnancy** | **Postpartum** | | | | | | | |
| --- | --- | --- | --- | --- | --- | --- | --- | --- | --- |
|  | **Antenatal visit** | **10**  **Weeks** | **6**  **Months** | **12 months** | **18 months** | **24 months** | **36 months** | **48 months** | **60 months** |
| IPV | N=869 | N=636 | N=613 | N=731 | N=655 | N=741 | N=769 | N=870 | N=830 |
|  | 295 (34.0%) | 165 (25.9%) | 177 (28.9%) | 199 (27.2%) | 143 (21.8%) | 157 (21.2%) | 139 (18.1%) | 105 (12.1%) | 76 (9.2%) |
| Maternal childhood trauma | N=869 |  |  |  |  |  |  |  |  |
|  | 296 (34.1%) | - | - | - | - | - | - | - | - |
| Alcohol use | N=862 | N=636 | N=610 | N=696 | N=629 | N=710 | N=747 | N=866 | N=825 |
|  | 141 (16.4%) | 98 (15.4%) | 128  (21.0%) | 178 (25.6%) | 190 (30.2%) | 211 (29.7%) | 243 (32.5%) | 301 (34.8%) | 268 (32.5%) |
| Stressful life events [Mean (SD)] | N=867 | N=636 | N=612 | N=697 | N=629 | N=716 | N=751 | N=872 | N=831 |
|  | 1.94  (2.22) | 1.30 (1.84) | 1.44 (2.06) | 1.41 (2.21) | 1.62 (2.12) | 1.72 (2.34) | 2.45 (2.60) | 2.17 (2.29) | 1.97 (2.32) |

**Supplementary Table 2.** Summary statistics of maternal psychosocial exposures over time in mothers included in analysis

IPV: Intimate partner violence; SD: Standard deviation

**Supplementary Table 3.** Unadjusted multinomial logistic regression model of predictors of the latent tracjectory classes of psychological distress using a complete case approach

|  | **Class 1 vs Class 4** | **Class 2 vs Class 4** | **Class 3 vs Class 4** |
| --- | --- | --- | --- |
|  | **Unadjusted**  **RRR**  **(95% CI)** | **Unadjusted**  **RRR**  **(95% CI)** | **Unadjusted**  **RRR**  **(95% CI)** |
| Age at enrolment | 1.01 (0.97; 1.06) | 0.96 (0.92; 1.00) | 1.02 (0.96; 1.08) |
| BMI | 0.96 (0.92; 1.00) | 0.98 (0.94; 1.02) | 0.98 (0.93; 1.03) |
| Houeshold income: <1000 ZAR | Reference | Reference | Reference |
| 1000-5000 ZAR | 0.84 (0.49; 1.43) | 0.82 (0.49; 1.36) | 0.68 (0.34; 1.37) |
| >5000 ZAR | 0.99 (0.45; 2.18) | 1.08 (0.52; 2.25) | 0.89 (0.32; 2.50) |
| Self-reported Employment | 0.71 (0.38; 1.30) | 0.97 (0.57; 1.66) | 0.86 (0.40; 1.85) |
| Educational achievement: |  |  |  |
| Did not complete secondary | Reference | Reference | Reference |
| Completed secondary or above | 0.69 (0.41; 1.18) | 0.63 (0.38; 1.06) | 0.64 (0.31; 1.32) |
| Marital status: Single | Reference | Reference | Reference |
| Married/co-habiting | 1.29 (0.79; 2.12) | 1.19 (0.74; 1.91) | 1.28 (0.67; 2.47) |
| Partner support : No support | Reference | Reference | Reference |
| Slight/moderate support | 0.39 (0.15; 1.03) | **0.21 (0.07; 0.58)** | 1.04 (0.12; 9.32) |
| Considerable/extreme support | **0.21 (0.09; 0.51)** | **0.22 (0.10; 0.50)** | 1.13 (0.15; 8.64) |
| Gravida | **1.23 (1.01; 1.52)** | 0.99 (0.80; 1.24) | 1.05 (0.78; 1.41) |
| HIV infection | **0.40 (0.18; 0.89)** | 1.00 (0.57; 1.76) | 1.84 (0.92; 3.67) |
| Gestational diabetes | **3.94 (1.04; 14.91)** | - | 2.34 (0.29; 18.98) |
| Asthma | 1.15 (0.14; 9.08) | 1.02 (0.13; 8.11) | - |
| Hypertension | 2.25 (0.91; 5.59) | 0.96 (0.29; 3.21) | 0.64 (0.09; 4.81) |
| Anaemia | **0.45 (0.21; 0.95)** | 1.04 (0.60; 1.81) | 0.64 (0.26; 1.55) |
| Smoking during pregnancy | **3.88 (2.34; 6.42)** | **1.70 (1.04; 2.77)** | 1.30 (0.64; 2.62) |
| Alcohol* | **1.04 (1.01; 1.07)** | **1.03 (1.01; 1.06)** | 1.02 (0.98; 1.06) |
| IPV | **3.24 (1.94; 5.40)** | **2.69 (1.63; 4.43)** | 0.80 (0.37; 1.75) |
| Maternal childhood trauma* | **1.07 (1.05; 1.08)** | **1.05 (1.04; 1.07)** | **1.04 (1.01; 1.07)** |
| Traumatic life events* | **1.46 (1.32; 1.61)** | **1.46 (1.33; 1.61)** | 1.14 (0.97; 1.33) |

RRR: Relative risk ratio; CI: Confidence interval; BMI: Body mass index; HIV: Human Immunodeficiency Virus; IPV: Intimate partner violence; Class 1: Persistent psychological distress symptoms; Class 2: Antenatal symptoms only; Class 3: Late onset psychological distress; Class 4: No psychological distress.

*Total scores for psychosical measures used

**Supplementary Table 4.** Adjusted multinomial logistic regression model of Predictors of the latent tracjectory classes of psychological distress using a complete case approach

|  | **Class 1 vs Class 4** | **Class 2 vs Class 4** | **Class 3 vs Class 4** |
| --- | --- | --- | --- |
|  | **Adjusted***  **RRR**  **(95% CI)** | **Adjusted***  **RRR**  **(95% CI)** | **Adjusted***  **RRR**  **(95% CI)** |
| Age at enrolment | 0.99 (0.92; 1.07) | 0.93 (0.86; 1.00) | 0.99 (0.90; 1.08) |
| BMI | 0.99 (0.94; 1.04) | 0.99 (0.95; 1.04) | 0.99 (0.93; 1.05) |
| Houeshold income: <1000 ZAR | Reference | Reference | Reference |
| 1000-5000 ZAR | 0.71 (0.36; 1.37) | 0.75 (0.38; 1.45) | 0.65 (0.28; 1.48) |
| >5000 ZAR | 0.80 (0.28; 2.29) | 0.89 (0.34; 2.31) | 0.64 (0.17; 2.39) |
| Self-reported Employment | 0.83 (0.38; 1.78) | 1.77 (0.89; 3.50) | 0.90 (0.35; 2.33) |
| Educational achievement: |  |  |  |
| Did not complete secondary | Reference | Reference | Reference |
| Completed secondary or above | 1.04 (0.52; 2.07) | 0.80 (0.42; 1.52) | 0.91 (0.40; 2.07) |
| Marital status: Single | Reference | Reference | Reference |
| Married/co-habiting | 1.14 (0.58; 2.25) | 1.55 (0.80; 3.02) | 1.5 (0.66; 3.64) |
| Partner support : No support | Reference | Reference | Reference |
| Slight/moderate support | 0.58 (0.19; 1.80) | 0.30 (0.09; 1.02) | 1.15 (0.12; 10.69) |
| Considerable/extreme support | **0.28 (0.10; 0.76)** | **0.34 (0.12; 0.95)** | 1.20 (0.15; 9.74) |
| Gravida | 1.24 (0.88; 1.76) | 1.06 (0.73; 1.54) | 0.99 (0.62; 1.60) |
| HIV infection | 0.60 (0.24;1.52) | 1.49 (0.73; 3.08) | **2.72 (1.21; 6.09)** |
| Gestational diabetes | **19.03 (3.24; 111.58)** | - | 10.68 (0.97; 117.00) |
| Asthma | 1.61 (0.18; 14.70) | 1.29 (0.14; 11.80) | - |
| Hypertension | 2.88 (0.87; 9.53) | 2.20 (0.59; 8.24) | 0.96 (0.11; 7.97) |
| Anaemia | 0.52 (0.22; 1.19) | 1.29 (0.67; 2.49) | 0.64 (0.25; 1.63) |
| Smoking during pregnancy | 1.78 (0.94; 3.38) | 0.77 (0.41; 1.45) | 1.09 (0.46; 2.58) |
| Alcohol** | 1.02 (0.99; 1.06) | 1.03 (0.99; 1.07) | 1.02 (0.98; 1.07) |
| IPV | 1.44 (0.78; 2.67) | 1.55 (0.86; 2.80) | 0.57 (0.25; 1.31) |
| Maternal childhood trauma** | **1.05 (1.03; 1.07)** | **1.03 (1.02; 1.05)** | **1.03 (1.01; 1.06)** |
| Traumatic life events** | **1.28 (1.14; 1.44)** | **1.35 (1.20; 1.51)** | 1.11 (0.93; 1.32) |

RRR: Relative risk ratio; CI: Confidence interval; BMI: Body mass index; HIV: Human Immunodeficiency Virus; IPV: Intimate partner violence; Class 1: Persistent psychological distress symptoms; Class 2: Antenatal symptoms only; Class 3: Late onset psychological distress; Class 4: No psychological distress.

*n=847

**Total scores for psychosical measures used

**Supplementary Table 5.** Postnatal risk factors associated with the latent tracjectory classes of psychological distress using a complete case approach

|  | **12 months*** | | | **24 months*** | | | **36 months*** | | | **48 months*** | | | **60 months*** | | |
| --- | --- | --- | --- | --- | --- | --- | --- | --- | --- | --- | --- | --- | --- | --- | --- |
|  | **Class 1 vs Class 4** | **Class 2 vs Class 4** | **Class 3 vs Class 4** | **Class 1 vs Class 4** | **Class 2 vs Class 4** | **Class 3 vs Class 4** | **Class 1 vs Class 4** | **Class 2 vs Class 4** | **Class 3 vs Class 4** | **Class 1 vs Class 4** | **Class 2 vs Class 4** | **Class 3 vs Class 4** | **Class 1 vs Class 4** | **Class 2 vs Class 4** | **Class 3 vs Class 4** |
|  | **Adjusted**  **RRR**  **(95% CI)** | **Adjusted**  **RRR**  **(95% CI)** | **Adjusted**  **RRR**  **(95% CI)** | **Adjusted**  **RRR**  **(95% CI)** | **Adjusted**  **RRR**  **(95% CI)** | **Adjusted**  **RRR**  **(95% CI)** | **Adjusted**  **RRR**  **(95% CI)** | **Adjusted**  **RRR**  **(95% CI)** | **Adjusted**  **RRR**  **(95% CI)** | **Adjusted**  **RRR**  **(95% CI)** | **Adjusted**  **RRR**  **(95% CI)** | **Adjusted**  **RRR**  **(95% CI)** | **Adjusted**  **RRR**  **(95% CI)** | **Adjusted**  **RRR**  **(95% CI)** | **Adjusted**  **RRR**  **(95% CI)** |
| HIV Infection | 0.41 (0.14; 1.21) | 1.63 (0.76; 3.49) | **4.01 (1.22; 13.24)** | 0.38 (0.13; 1.12) | 1.46 (0.68; 3.16) | **3.31 (1.33; 8.21)** | **0.12 (0.03; 0.57)** | 0.69 (0.29; 1.68) | 1.55 (0.48; 4.96) | 0.44 (0.18; 1.08) | 1.44 (0.73; 2.86) | 2.07 (0.86; 4.97) | 0.52 (0.22; 1.23) | 1.17 (0.61; 2.24) | 1.22 (0.47; 3.16) |
| Child sex - female | 0.80 (0.41; 1.57) | 1.08 (0.57; 2.05) | 1.80 (0.62; 5.19) | 0.86 (0.43; 1.71) | 0.80 (0.42; 1.53) | 1.14 (0.50; 2.59) | 0.80 (0.40; 1.60) | 0.76 (0.40; 1.44) | 2.29 (0.79; 6.67) | 0.98 (0.56; 1.72) | 0.90 (0.52; 1.55) | 1.54 (0.70; 3.39) | 0.95 (0.55; 1.62) | 1.01 (0.60; 1.70) | 1.41 (0.63; 3.17) |
| Preterm delivery | 1.25 (0.52; 2.99) | 0.85 (0.34; 2.12) | 0.95 (0.25; 3.63) | 0.87 (0.34;1.24) | 0.48 (0.14; 1.63) | 0.87 (0.27; 2.80) | 0.74 (0.27; 2.00) | 0.32 (0.09; 1.11) | 0.97 (0.25; 3.74) | 1.12 (0.53; 2.37) | 0.51 (0.20; 1.32) | 0.95 (0.34; 2.67) | 0.98 (0.46; 2.12) | 0.79 (0.36; 1.75) | 0.88 (0.28; 2.82) |
| Child birth weight z-score | 0.83 (0.61; 1.14) | 0.87 (0.64; 1.17) | 1.07 (0.64; 1;79) | 0.82 (0.59; 1.14) | 0.88 (0.65; 1.19) | 1.20 (0.80; 1.82) | 0.97 (0.70; 1.350 | 1.20 (0.88; 1.64) | 1.09 (0.67; 1.78) | 0.82 (0.63; 1.07) | 1.00 (0.77; 1.31) | 1.09 (0.75; 1.59) | 0.83 (0.64; 1.07) | 1.07 (0.83; 1.37) | 1.22 (0.83; 1.81) |
| Houeshold income: | |  |  |  |  |  |  |  |  |  |  |  |  |  |  |
| <1000 ZAR | Reference | Reference | Reference | Reference | Reference | Reference | Reference | Reference | Reference | Reference | Reference | Reference | Reference | Reference | Reference |
| 1000-5000 ZAR | **0.36 (0.16; 0.84)** | 0.72 (0.29; 1.79) | 0.43 (0.11; 1.59) | 1.04 (0.37; 2.90) | 1.33 (0.42; 4.21) | 0.72 (0.23; 2.27) | 1.05 (0.42; 2.62) | 1.40 (0.49; 3.96) | 0.41 (0.12; 1.45) | 0.85 (0.42; 1.74) | 3.38 (0.98; 11.63) | 0.76 (0.27; 2.18) | 0.93 (0.42; 2.07) | 1.62 (0.54; 4.87) | 0.44 (0.15; 1.28) |
| >5000 ZAR | 0.37 (0.11; 1.21) | 1.32 (0.42; 4.20) | 1.87 (0.38; 9.17) | 0.54 (0.14; 2.03) | 1.17 (0.32; 4.32) | 1.14 (0.31; 4.25) | 0.57 (0.18; 1.79) | 1.31 (0.42; 4.05) | 0.72 (0.18; 3.00) | **0.37 (0.15; 0.91)** | 2.70 (0.73; 9.95) | 0.85 (0.26; 2.78) | 1.28 (0.54; 3.00) | 1.99 (0.63; 6.26) | 0.58 (0.18; 1.93) |
| Self-reported Employment | 0.51 (0.23; 1.14) | 1.25 (0.64; 2.44) | 0.80 (0.25; 2.57) | 0.50 (0.23; 1.08) | 1.16 (0.59; 2.30) | 0.96 (0.38; 2.45) | 0.89 (0.42; 1.89) | 1.36 (0.69; 2.67) | 0.37 (0.12; 1.15) | 0.68 (0.37; 1.25) | 1.68 (0.92; 3.05) | 1.02 (0.45; 2.32) | **0.40 (0.22; 0.74)** | 1.74 (0.99; 3.04) | 1.08 (0.44; 2.65) |
| Educational achievement: |  |  |  |  |  |  |  |  |  |  |  |  |  |  |  |
| Did not complete secondary | Reference | Reference | Reference | Reference | Reference | Reference | Reference | Reference | Reference | Reference | Reference | Reference | Reference | Reference | Reference |
| Completed secondary or above | 0.84 (0.39; 1.80) | 0.65 (0.30; 1.40) | 0.70 (0.20; 2.43) | 1.43 (0.67; 3.06) | 0.93 (0.46; 1.91) | 0.96 (0.38; 2.45) | **0.39 (0.17; 0.91)** | **0.44 (0.21; 0.93)** | 1.49 (0.51; 4.32) | 0.91 (0.49; 1.69) | 0.76 (0.41; 1.38) | 0.73 (0.30; 1.74) | 0.84 (0.47; 1.52) | 0.65 (0.37; 1.16) | 0.69 (0.28; 1.69) |
| Marital status: Single | Reference | Reference | Reference | Reference | Reference | Reference | Reference | Reference | Reference | Reference | Reference | Reference | Reference | Reference | Reference |
| Married/co-habiting | 0.91 (0.45; 1.83) | 1.11 (0.58; 2.11) | 0.93 (0.32; 2.65) | 1.31 (0.66; 2.62) | 0.89 (0.47; 1.71) | 0.48 (0.20; 1.13) | 0.50 (0.24; 1.03) | 0.70 (0.36; 1.34) | 1.02 (0.37; 2.81) | 1.09 (0.61; 1.96) | 0.92 (0.52; 1.62) | 1.36 (0.60; 3.09) | 0.83 (0.48; 1.44) | 1.01 (0.59; 1.74) | 0.84 (0.36; 1.95) |
| Smoking | 1.26 (0.62; 2.61) | 1.38 (0.67; 2.86) | 1.12 (0.34; 3.67) | 1.98 (0.96; 4.09) | 1.84 (0.90; 3.75) | 1.63 (0.63; 4.23) | **2.23 (1.06; 4.68)** | **2.24 (1.12; 4.48)** | 1.48 (0.51; 4.34) | **2.62 (1.43; 4.81)** | **2.02 (1.08; 3.76)** | 1.57 (0.65; 3.78) | **2.21 (1.23; 3.96)** | 1.71 (0.96; 3.05) | 1.57 (0.66; 3.76) |
| Alcohol** | 1.00 (0.97; 1.04) | 0.96 (0.91; 1.01) | 1.03 (0.99; 1.09) | 0.99 (0.95; 1.03) | 0.93 (0.86; 1.00) | 0.96 (0.89; 1.03) | 1.03 (0.99; 1.07) | 0.98 (0.93; 1.03) | 1.00 (0.94; 1.06) | 0.96 (0.91; 1.00) | 0.96 (0.92; 1.01) | 0.98 (0.93; 1.03) | 1.00 (0.96; 1.03) | **0.95 (0.90; 1.00)** | 1.01 (0.97; 1.06) |
| IPV | **4.57 (2.23; 9.38)** | 1.17 (0.55; 2.50) | **3.81 (1.26; 11.53)** | **5.61 (2.74; 11.46)** | 1.26 (0.54; 2.92) | **3.19 (1.27; 8.01)** | 2.00 (0.94; 4.29) | 1.38 (0.59; 3.25) | **3.88 (1.38; 10.89)** | 1.82 (0.88; 3.73) | 1.09 (0.46; 2.61) | **5.00 (2.22; 11.27)** | 1.21 (0.51; 2.85) | 1.20 (0.47; 3.06) | **3.51 (1.30; 8.81)** |
| Traumatic life events** | **1.36 (1.20; 1.55)** | 1.13 (0.98; 1.31) | 1.02 (0.81; 1.29) | **1.28 (1.14; 1.43)** | 0.95 (0.81; 1.13) | 0.95 (0.78; 1.16) | **1.25 (1.12; 1.41)** | 1.04 (0.92; 1.18) | **1.18 (1.01; 1.37)** | **1.21 (1.08; 1.35)** | **1.14 (1.00; 1.29)** | **1.27 (1.09; 1.48)** | **1.19 (1.07; 1.33)** | **1.15 (1.03; 1.28)** | **1.47 (1.28; 1.69)** |

RRR: Relative risk ratio; CI: Confidence interval; HIV: Human Immunodeficiency Virus; IPV: Intimate partner violence; Class 1: Persistent psychological distress symptoms; Class 2: Antenatal symptoms only; Class 3: Late onset psychological distress; Class 4: No psychological distress.

*12 month postpartum model n=562; 24 month postpartum model n=591; 36 month postpartum model n=616; 48 month postpartum model n=820; 60 month postpartum model n=816

**Total scores for psychosical measures used
